# Invasive pulmonary aspergillosis with brain dissemination in an immunocompetent host

**DOI:** 10.4322/acr.2021.280

**Published:** 2021-05-06

**Authors:** Senthil Kumar, Valliappan Muthu, Yogender Singh Bansal, Nikhil Mehta, Vanshika Arora

**Affiliations:** 1 Postgraduate Institute of Medical Education and Research (PGIMER), Department of Forensic Medicine, Chandigarh, India; 2 Postgraduate Institute of Medical Education and Research (PGIMER), Department of Pulmonary Medicine, Chandigarh, India

**Keywords:** Invasive Pulmonary Aspergillosis, Neuroaspergillosis, Autopsy, Microscopy

## Abstract

Invasive aspergillosis is an uncommon infection, which is mainly seen among immunocompromised patients. In recent years, cases of aspergillosis involving immunocompetent hosts are increasingly being reported. Herein, we report the case of a 27-year-old man with fever, productive cough, shortness of breath, and left hemiparesis. He had suffered trauma to his head 25 days prior. Imaging of the chest showed bilateral cavitary lesions in the lungs, and neuroimaging revealed a space-occupying lesion in the right frontoparietal cerebrum. He was suspected of having an abscess or metastasis. He died on day 3 of hospitalization, and an autopsy was performed. The autopsy revealed the cause of death to be invasive pulmonary aspergillosis, with brain dissemination. Invasive aspergillosis is uncommon in apparently immunocompetent individuals, and we discuss the autopsy findings in detail.

## INTRODUCTION

Aspergillosis is an infection caused by a fungus of the genus *Aspergillus*, which is ubiquitous.^1^ Depending on the host immune status, *Aspergillus* can cause a variety of diseases, including allergic reactions, invasive lung infections, and infections of other organs.^2^ The primary sites of infection are the lungs and paranasal sinuses. Cerebral involvement arises from direct invasion from the paranasal sinuses or by hematogenous spread.^3^ Invasive aspergillosis is a cause of significant morbidity and mortality in severely immunocompromised hosts, but is rarely reported among immunocompetent hosts.^1^ Here we report the case of invasive aspergillosis involving the lungs and brain in an immunocompetent person.

## CASE REPORT

A 27-year-old-male was allegedly beaten on the head with a wooden stick, following which he was immediately taken to a local civil hospital. There was no history of loss of consciousness, vomiting, seizure, or bleeding from the ear, nose, or throat. On medicolegal examination, an injury over the forehead’s left side was noted, and the patient was discharged after first aid.

**Figure 1:**
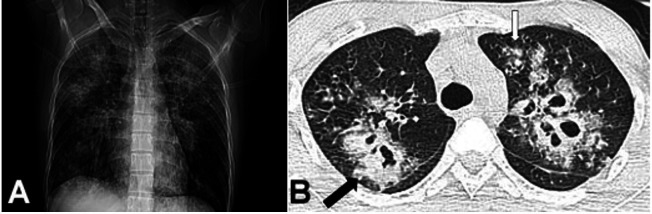
**A** – Chest x-ray showing bilateral heterogeneous opacities in the pulmonary parenchyma; **B** – Thoracic computed tomography showing bilateral upper lobe nodules of varying size and multiple cavitations. One of the them shows intracavity content (black arrow). Ground glass opacities surrounding a few nodules in the left upper lobe (halo sign; white arrow).

Approximately 25 days after the alleged assault, the patient was brought to the outpatient department complaining of fever, productive cough, and shortness of breath for the past 15 days, and weakness on the left side of his body. He did not have any ear pain, discharge, or nose block. He denied any history of recurrent diarrhea or respiratory infections since childhood. He was not a smoker or drug abuser and did not have any past respiratory illness. He had been working at a construction site for 3 months before the illness. On examination, his Glasgow Coma Scale (GCS) score was 15, and the central nervous system examination revealed left hemiparesis. Non-contrast brain computed tomography showed hypodensity in the right frontal and parietal region adjacent to the ventricle. A chest x-ray showed bilateral heterogeneous infiltration of the lungs (Figure 1A). A high-resolution chest computed tomography showed bilateral nodules of varying size and multiple cavities. The abnormalities were evident in both lungs, mainly in the bilateral upper lobes. There was no pleural effusion or significant mediastinal lymphadenopathy (Figure 1B). He was managed with broad-spectrum antibiotics. The diagnostic possibility of tuberculosis was considered, and empirical anti-tuberculosis treatment was started. Five days later, his GCS score deteriorated to 7 and he was admitted to the ward. Cerebral spinal fluid analysis showed 2,100 cells (reference range [RR]: 0–5 cells/mm^3^) with a predominance of lymphocytes, the glucose of 95 mg/dL (RR: 50–80 mg/dL), proteins of 16 mg/dL (RR: 15–40 mg/dL). Serological investigations for HIV, and hepatitis B and C were negative. A hemogram showed a total leucocyte count of 10,300/mm^3^ and a differential count was within normal limits; the hemoglobin was 14.4 mg/dL (RR; 13-15 g/dL) and the platelet count was 219,000/μL (RR 150,000-450,000/ μL). His liver function tests and renal function tests were within normal limits. On the third day of hospitalization, he succumbed to the illness. The autopsy was performed owing to the legal implications of this patient's case.

## AUTOPSY FINDINGS

**Figure 2:**
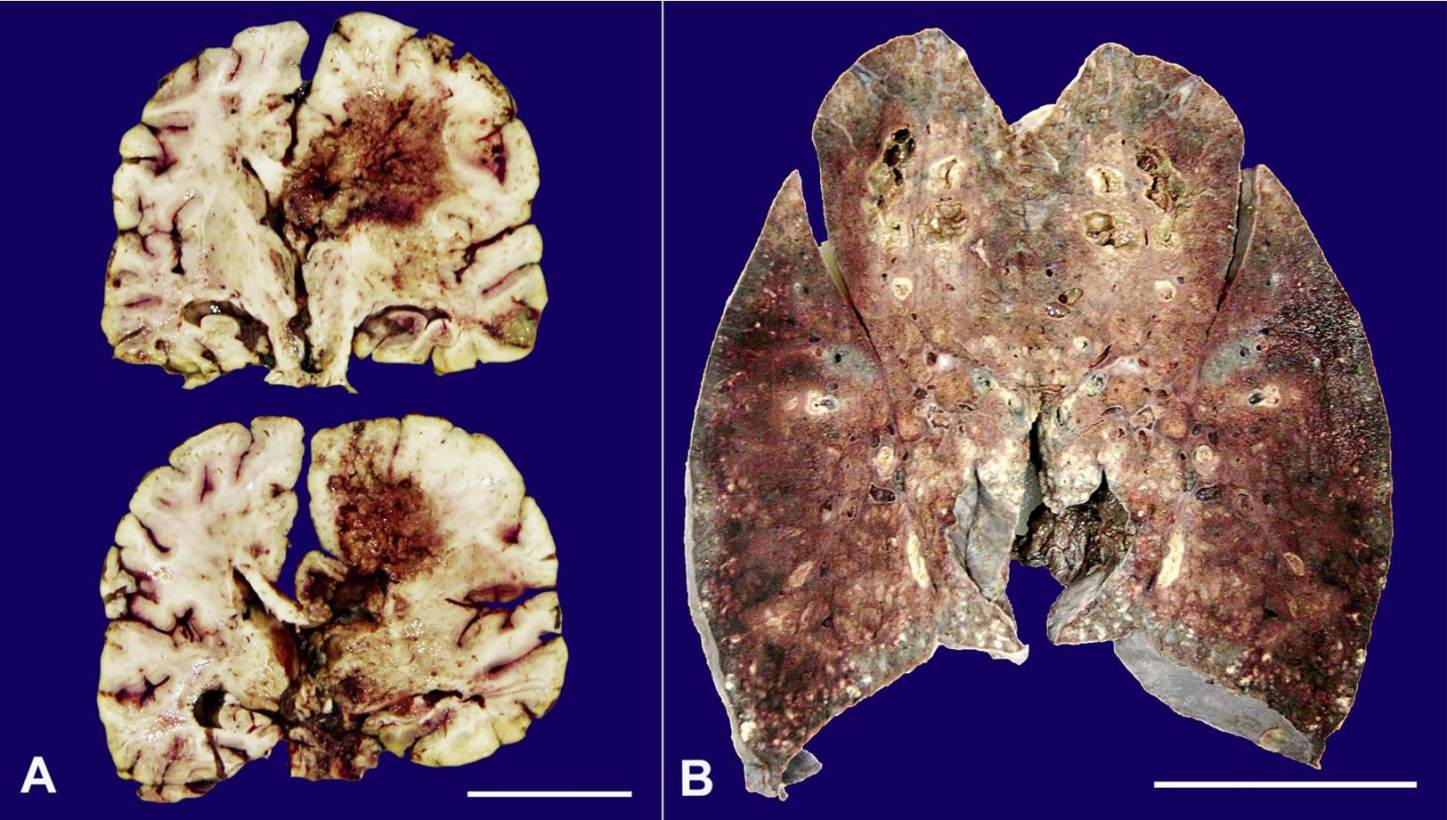
**A** – Cut section of formalin-fixed brain: Hemorrhagic necrotic area in the right frontal and parietal lobes involving corpus callosum, caudate nucleus, lateral ventricle, and thalamus, an area of purulent exudates and necrosis in the cerebral cortex of the right temporal lobe; **B** – Cut section of formalin-fixed left lung: Multiple yellowish-white nodules, cavitary lesions filled with necrotic material, and areas of hemorrhagic necrosis.

On external examination, the deceased was 173 cm in length and of average build. We observed a linear scar of 6.5 cm vertically over the left side of the forehead and the scalp’s frontal region. On reflecting the scalp, the scar was seen extending up to the periosteal layer of the skull bone. No underlying skull fracture was seen. There was no intracranial hemorrhage on internal examination; the brain was edematous weighing 1600 g (RR; 1075-1685g) and no basal exudates were seen. On the brain cut section, we noted an area of hemorrhagic necrosis in the right frontal and parietal lobes involving the corpus callosum, basal nuclei, lateral ventricle, and thalamus. The midline shift and compression of the adjacent brain parenchyma were noted. An area of necrosis with purulent exudate was seen in the cerebral cortex of the right temporal lobe (Figure 2A). Both lungs were enlarged and of hard consistency. The right lung weighed around 1050 g (RR: 360–570 g) and the left lung 1000 g (RR: 325–480 g). Multiple yellowish-white nodules were present on the surface and the cut section, and some nodules showed necrosis (Figure 2B). The hilar lymph nodes were slightly enlarged, and extensive consolidation was present in both lungs. Some cavitary lesions filled with necrotic material were present in the upper lobes of both the lungs. No pulmonary embolism or lung infarction could be seen. The heart and coronary arteries were grossly normal; the liver was enlarged, congested, and weighed 1730 g (mRR; 1540 g); the spleen and both kidneys were grossly normal except for some congestion.

**Figure 3:**
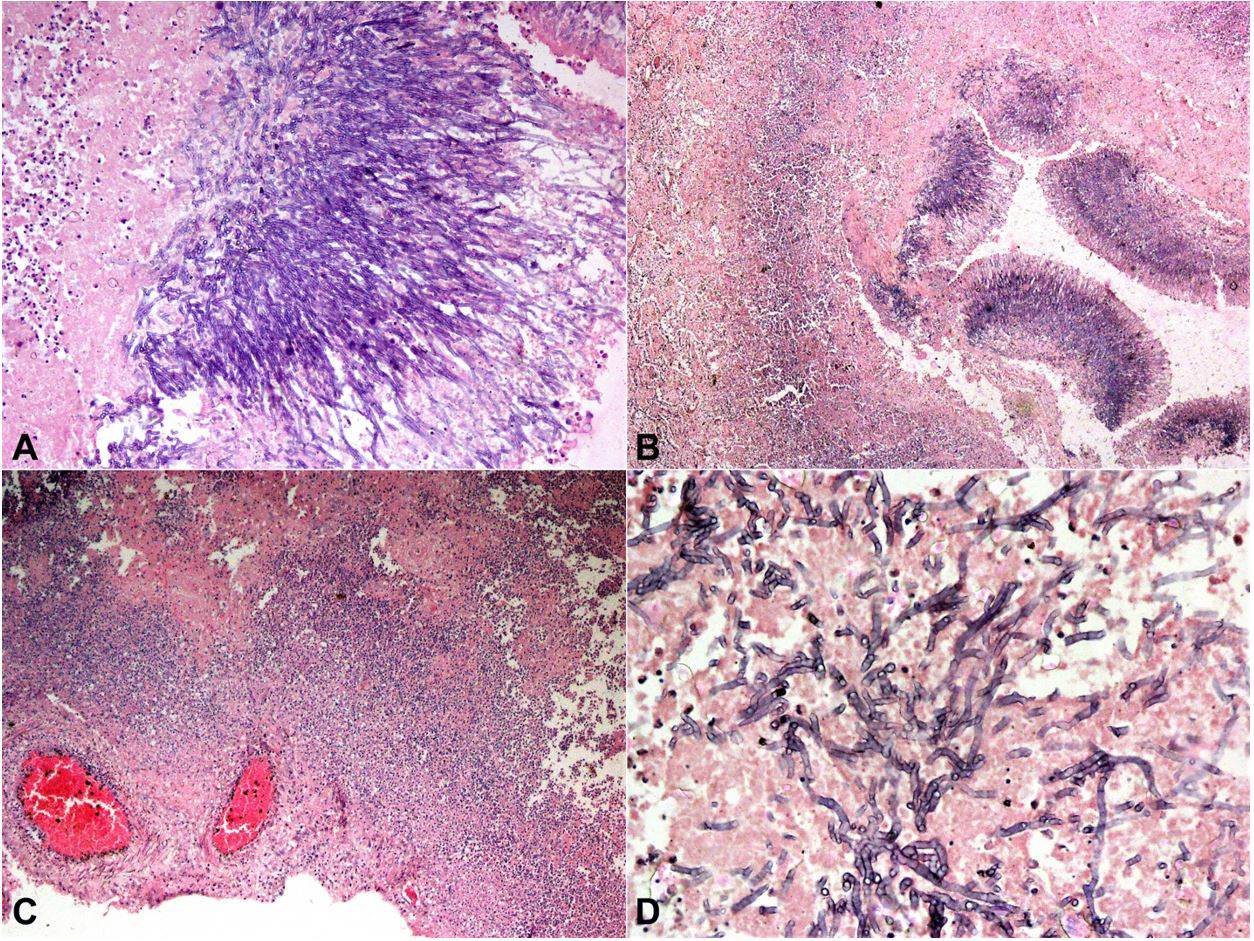
**A** and **B** – Photomicrographs of the lungs showing fungal abscess (blue staining), acutely branching septate hyphal elements of *Aspergillus* spp. with heavy infiltration of neutrophils with the background of necrotic lung tissues (H&E A 10X, and B 4X); **C** and **D** – Photomicrographs of the brain; **C** – showing necrosis, heavy neutrophil infiltration with thrombosed vessels (H&E, 4X); **D** – Multiple blue-stained septate fungal hyphae (H&E, 40X).

On microscopic examination, sections from the lungs showed a fungal abscess showing blue staining, acutely branching septate hyphal elements consistent with the morphology of *Aspergillus* spp. (Figure 3A). We also noted the heavy infiltration of neutrophils in the necrotic lung tissues (Figure 3B). A section also showed a cavity filled with a fungus ball and heavy neutrophilic infiltration. Some thrombosed vessels with the invasion of fungal elements were noted. Acid-fast bacillus staining was negative, and Grocott’s silver stain showed black hyphae of *Aspergillus* invading the lung tissue (Figure 44B). Multiple brain sections showed foci of hemorrhagic necrosis along with heavy infiltration of neutrophils, and perivascular infiltration of inflammatory cells (Figure 3C). Multiple, blue-stained, septate fungal hyphae were identified on routine hematoxylin and eosin stain (Figure 3D). Some sections showed brain edema and thrombosed vessels. Sections from the liver showed intact liver architecture with sinusoidal dilatation (shock-related changes). Spleen and kidney sections showed congestion. The lymph nodes were normal with preserved architecture.

**Figure 4:**
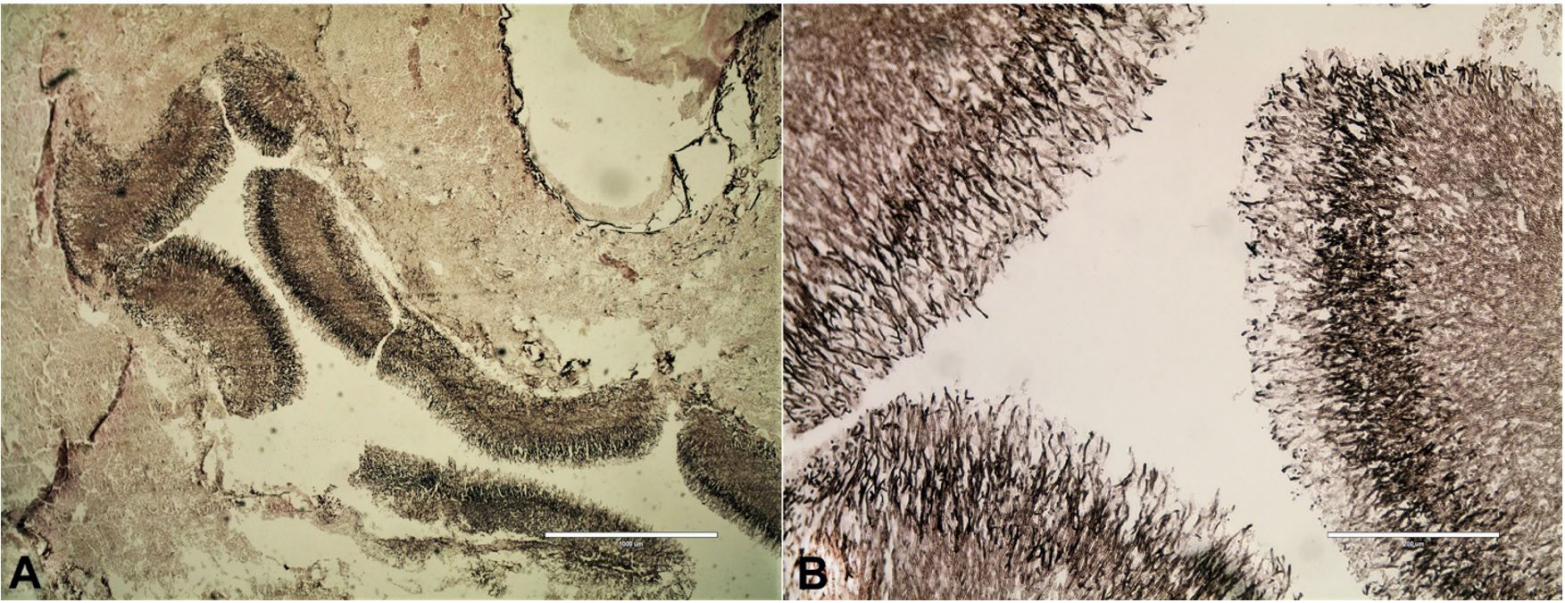
Photomicrograph of the brain. **A** and **B** – show acutely branching septate hyphal elements of aspergillus (Grocott’s staining A 4X, and B 10X).

## DISCUSSION

Disseminated aspergillosis in an immunocompetent host is uncommon, and autopsy descriptions are limited. Herein, we have described a case of disseminated aspergillosis in an apparently immunocompetent 27-year-old man. The diagnosis was not suspected in life, and he succumbed to the illness. There is a gap in the knowledge and understanding of the clinical features, the progression of the disease process, and the treatment prognosis in immunocompetent individuals. Therefore, the diagnosis of fungal infection in immunocompetent patients is often missed or delayed.^4^

Moreover, as the clinical presentation of invasive pulmonary aspergillosis (IPA) usually mimics tuberculosis, the patients often receive antitubercular therapy, despite the lack of microbiologic confirmation, especially in high tubercular burden countries like India.^5^ While certain radiologic features, such as the presence of thick-walled cavity and halo sign, would have pointed towards invasive aspergillosis, culture or histopathological examination is essential. Sputum examination and bronchoalveolar lavage fluid examination could have assisted clinicians to reach the precise diagnosis. However, the rapid progression of the disease course, in the index case, left only a narrow window for making the diagnosis. Further, some of these patients may be unfit for a bronchoscopy procedure and in such scenarios, an elevated serum galactomannan can point towards the diagnosis. Diagnosing fungal infections is challenging, especially in immunocompetent individuals.^6^ In the absence of an apparent host factor in the index case, a biopsy was essential to confirm the diagnosis. Finally, the autopsy and histopathological examination revealed multiple fungal abscesses in the lungs with angioinvasion and brain dissemination. Pulmonary hilar lymph nodes were normal in architecture with no cell depletion, and there were no other apparent microscopic findings suggestive of chronic granulomatous disease or immune suppression. However, we have not evaluated the neutrophil function in the index case by performing the dihydrorhodamine test or the nitroblue tetrazolium test. The literature says that clinically, about 40% of invasive aspergillosis is missed and diagnosed only on autopsy.^2^ Therefore, a high index of suspicion is needed to diagnose aspergillosis, especially in immunocompetent patients.

*Aspergillus*, being ubiquitous, is the most common fungus infecting human beings. Among the various species, it is the “*Aspergillus fumigatus*” that most commonly produces disease by primarily infecting the lungs. The main portal of entry is by inhalation of fungal spores.^1^ In the majority of cases, it is the immunocompromised individuals who become the victims of such fungal infection. The profound neutropenia in immunosuppressed individuals can result in IPA. Nevertheless, in construction sites the abundant exposure of spores may cause disease even in the immunocompetent host.^2^ The patient in the index case worked in a construction site as a security guard, which explains the possible infection source, despite his apparent immunocompetent status. According to the history from his next-of-kin, the deceased resided in the same house for several years and he was not suffering from any chronic illness. As the head trauma was only mild without any skull fracture or intracranial hemorrhage, we could not associate it with the disseminated aspergillosis. The workplace insult seemed to be the only source of infection in this case, and we could not find any other source in his environment.

The extra-pulmonary dissemination generally occurs in the severely immunocompromised host. Such dissemination usually occurs via hematogenous spread from the primary infection site in the lungs and infected heart valves.^7^ The brain invasion is comparatively the more common entity of extra-pulmonary aspergillosis. The fungal hyphae invade the vessel wall obstructing cerebral blood flow resulting in infarction areas, which are usually hemorrhagic. The most frequently involved areas of the brain are the basal nuclei, thalamus, and splenium of the corpus callosum. Aspergillus abscess can be evident at the grey-white matter junction.^8^ The index case showed a lesion of hemorrhagic necrosis in the right frontoparietal lobe involving basal nuclei, thalamus, and corpus callosum, and a small fungal abscess in the right temporal lobe at the grey-white matter junction.

## CONCLUSION

Disseminated aspergillosis is uncommon and continues to remain unsuspected in immunocompetent individuals. IPA is frequently misdiagnosed as tuberculosis or pneumonia due to non-specific manifestations like productive cough, fever, and dyspnea in the early phase of the infection. The radiologic findings are also non-specific. Culture, molecular diagnosis, and histopathological evaluation of fungal etiology should be attempted in such cases. A high index of suspicion and timely diagnosis with early intervention can prevent the dissemination and improve the outcome in an immunocompetent host with aspergillus infection.

## References

[1] El Hasbani G, Chirayil J, Nithisoontorn S (2019). Cerebral aspergillosis presenting as a space occupying lesion in an immunocompetent individual. Med Mycol Case Rep.

[2] Jameson JL, Denning DW (2018). Aspergillosis.. Harrison’s principles of internal medicine..

[3] Narayan SK, Kumar K, Swaminathan RP, Roopeshkumar VR, Bhavna B (2009). Isolated cerebral aspergilloma in a young immunocompetent patient. Pract Neurol.

[4] Xu X-Y, Sun H-M, Zhao B-L, Shi Y (2013). Diagnosis of airway-invasive pulmonary aspergillosis by tree-in-bud sign in an immunocompetent patient: case report and literature review. J Mycol Med.

[5] Kim SH, Kim MY, Hong SI (2015). Invasive pulmonary aspergillosis-mimicking tuberculosis. Clin Infect Dis.

[6] Haydour Q, Hage CA, Carmona EM (2019). Diagnosis of fungal infections. A systematic review and meta-analysis supporting American Thoracic Society Practice Guideline. Ann Am Thorac Soc.

[7] Li W, Shafi N, Periakaruppan R, Valyi-Nagy T, Groth J, Testai FD (2015). Cerebral aspergillosis in a diabetic patient leading to cerebral artery occlusion and ischemic stroke: a case report and literature review. J Stroke Cerebrovasc Dis.

[8] Sidani C, Freiser ME, Saigal G, Sklar E (2013). Unusual case of cerebral aspergillosis with clinical and imaging findings mimicking lymphoma. Neuroradiol J.

